# Beware of covert enemies: *Candida orthopsilosis* malignant otitis externa with base of the skull osteomyelitis, a case report and review of literature

**DOI:** 10.1016/j.idcr.2021.e01163

**Published:** 2021-05-18

**Authors:** Junais Koleri, Ahmad Al Bishawi, Israa' Al-Sheikh, Salman Qureshi, Muna AlMaslamani, Hamad Abdelhadi

**Affiliations:** a Department of Infectious Diseases, Communicable Diseases Centre, Hamad Medical Corporation, Qatar; b Department of Internal Medicine, Hamad Medical Corporation, Qatar; c Department of Neuroradiology, Hamad Medical Corporation, Qatar

**Keywords:** Malignant otitis externa, MOE, *Candida orthopsilosis*, Osteomyelitis

## Abstract

**Background:**

Malignant otitis externa (MOE) is a serious infection of the external auditory canal that is frequently associated with skull base osteomyelitis (SBO) as well as secondary neurological sequelae. Patients with poorly controlled diabetes mellitus or immunosuppression are at increased risk of developing such critical infection for multiple local and systemic factors. While most cases are secondary to bacterial infections particularly*Pseudomonas aeruginosa*, fungal infections are also occasionally encountered, often associated with delayed diagnosis and high morbidity and mortality.

**Case report:**

We report a case of a 63 years old man with uncontrolled diabetes mellitus who presented with symptoms and signs of MOE, supported by radiological assessments. The patient was treated presumptively with a prolonged course of antibiotics without clinical improvement, coupled with progression of radiological findings and significant disease extension. Reassessment with biopsies and tissue cultures from external auditory meatus, tempo-mandibular bone, as well as base of the skull grew *Candida orthopsilosis*. The patient received induction treatment with high dose liposomal amphotericin followed by fluconazole to control disease progression and complications.

**Conclusion:**

*Candida* MOE with secondary skull base osteomyelitis is rare and difficult to diagnose with no clear guidance on assessment and management. Clinicians should be aware of the unusual presentations where microbiological and histopathological evaluations are essential for proper management.

## Case report

A 63 years old man presented to our emergency department with a right sided headache and vertigo of one week duration along with right sided decreased hearing. There was no ear discharge. Review of systems were unremarkable. The past medical history was significant for a long-standing type 2 diabetes mellitus with secondary complications of retinopathy, dyslipidemia, and systemic hypertension. Drug history included insulin in addition to linagliptin, perindopril, and rosuvastatin. The patient has no history of smoking or alcohol consumption. Family history included diabetes mellitus and hypertension.

Vital signs were within normal limits. Physical examination was unremarkable apart from partial left sixth cranial nerve palsy with no associated nystagmus or other cerebellar signs. Local examination of the ears, nose, and throat showed no significant tenderness, color change, or pre or postauricular swelling in affected ear. Tympanic membranes were intact. Neck and head examination revealed no associated swellings or palpable lymph nodes, and the temporomandibular joint were normal.

**Fig. 1:**
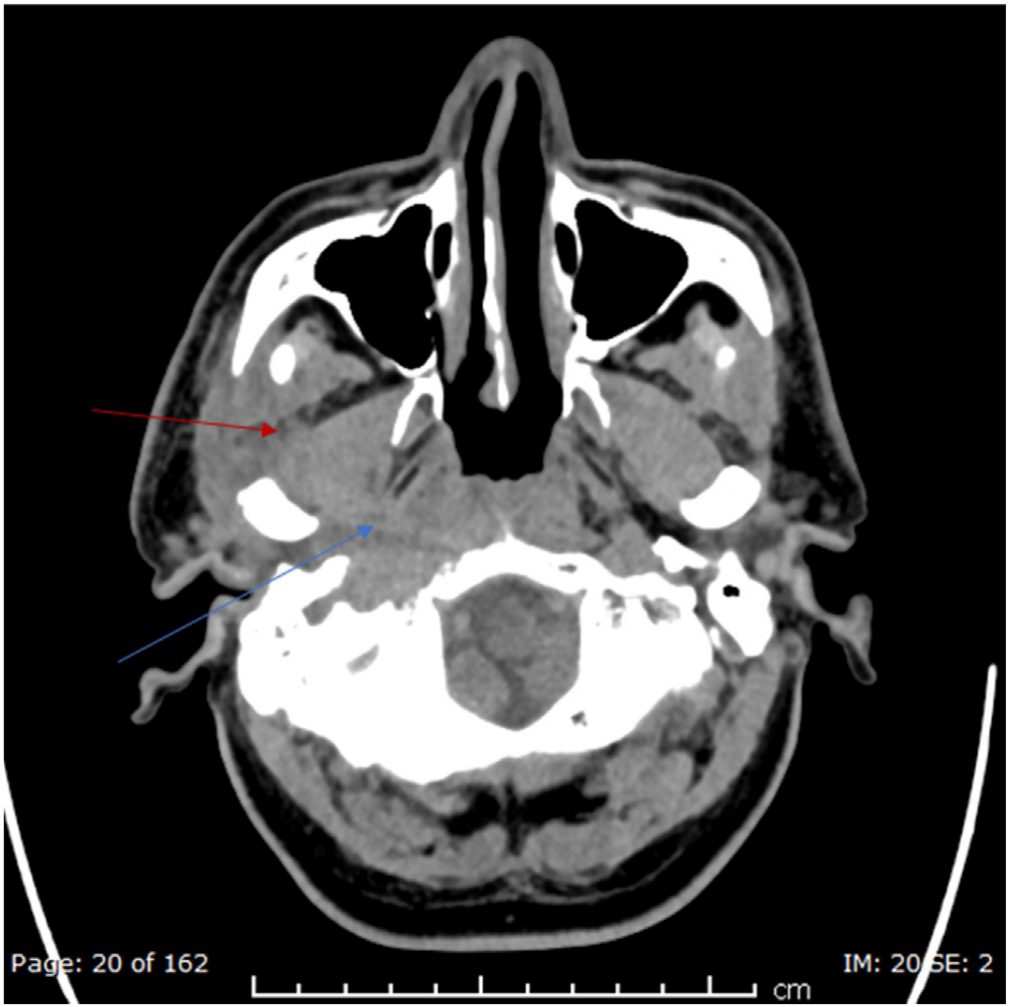
Non contrast axial CT initial scan in the emergency room demonstrated no significant intracranial pathology. However the soft tissue windows demonstrated loss of fat planes in the right parapharyngeal space (blue arrow) and masticator space (red arrow). (For interpretation of the references to colour in this Figure legend, the reader is referred to the web version of this article).

**Fig. 2:**
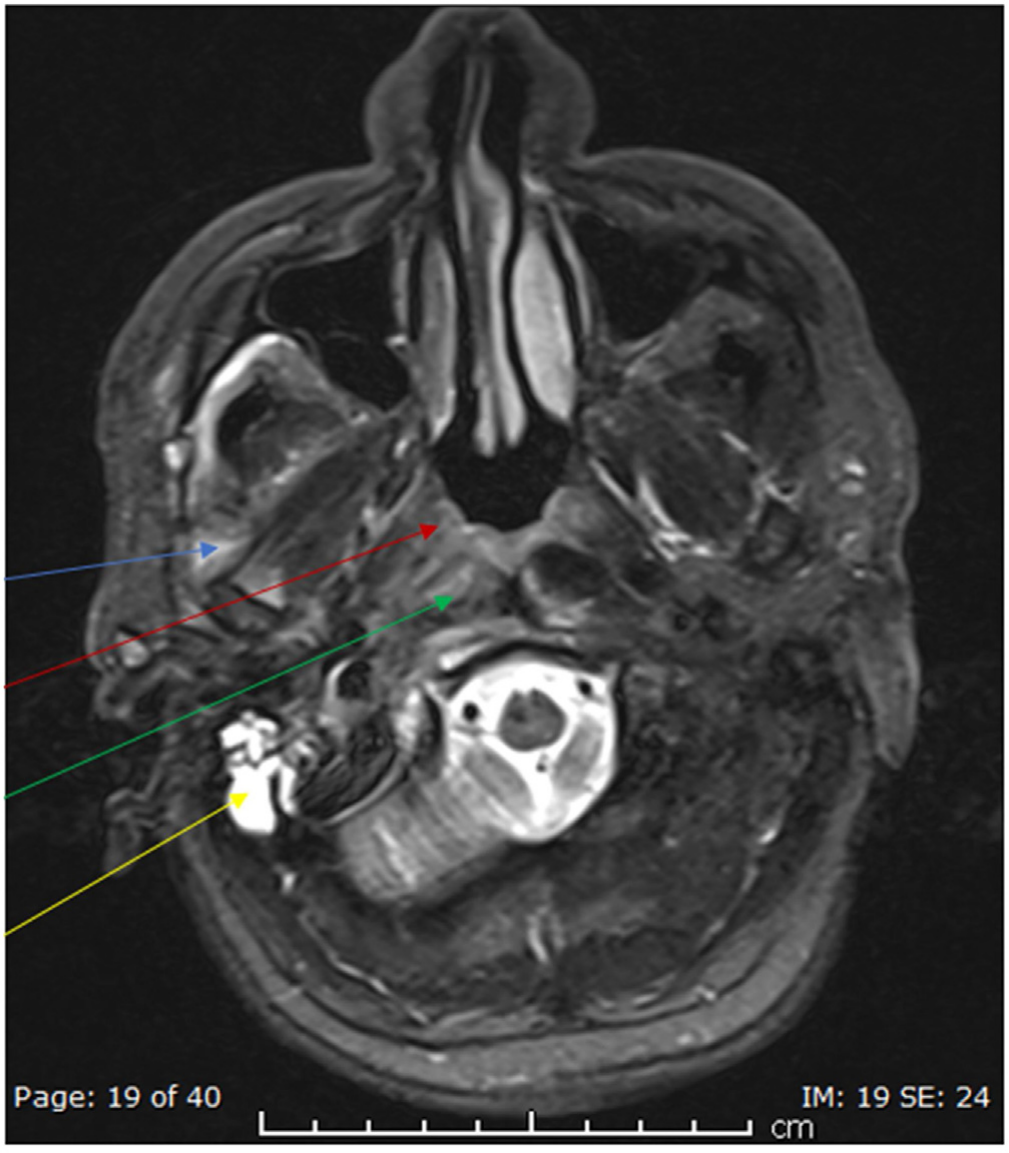
Axial STIR MRI sequence demonstrates hyperintensity (oedema) in multiple neck spaces including masticator space adjacent to the lateral pterygoid muscle (blue arrow). There is mucosal congestion within the nasopharynx (red arrow) with high signal noted posteriorly in the longus capitis muscle (green arrow) suggestive of extension through the retropharyngeal space. Note fluid secretions within the right mastoid air cells (yellow arrow). (For interpretation of the references to colour in this Figure legend, the reader is referred to the web version of this article).

**Fig. 3:**
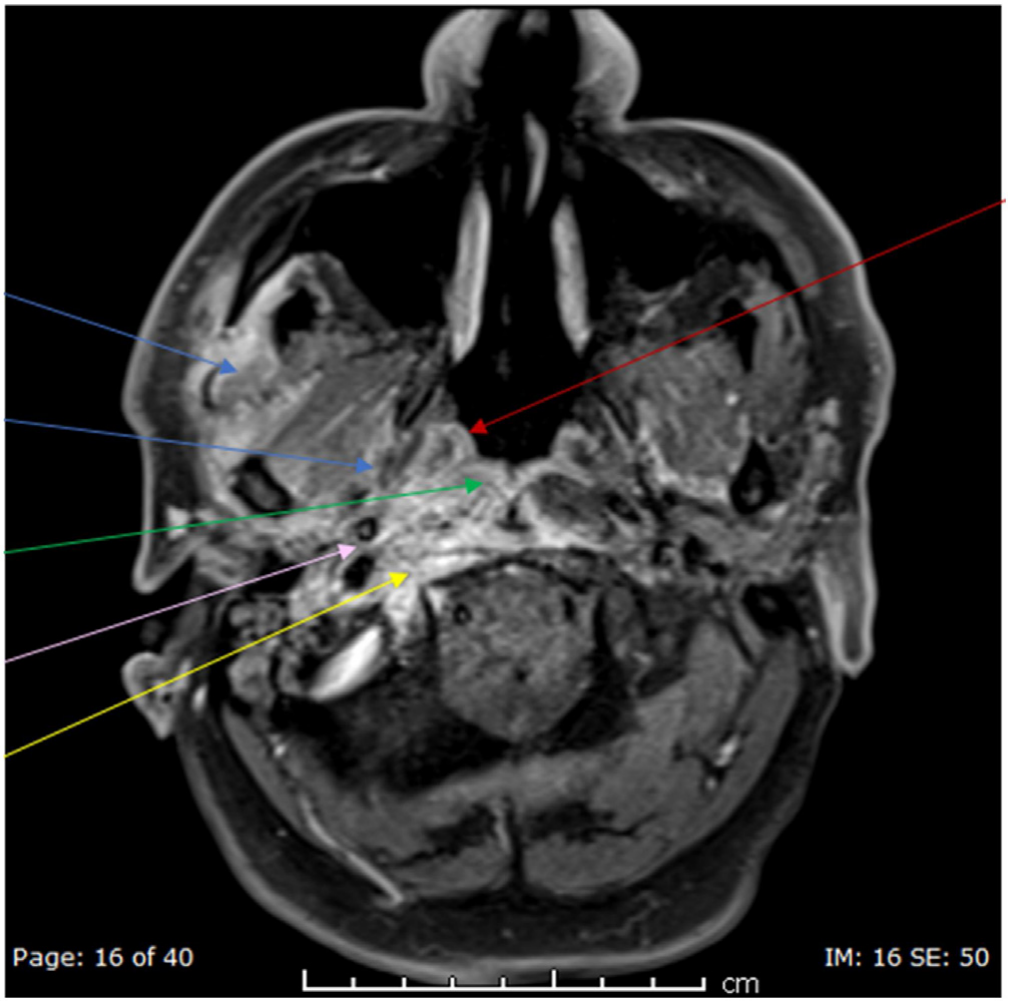
Axial and coronal post contrast enhanced MRI scan confirming diffuse multicompartmental involvement with enhancement in the masticator and parapharyngeal spaces as described previously (blue arrows). In addition, there is better delineation of medial and posterior extension including pharyngeal mucosal space of the nasopharynx (red arrow), retropharyngeal space (green arrow) and perivertebral space (yellow arrow). Note the enhanacment in the right carotid space surrounding IJV and ICA (pink arrow). Coronal image delineates extension into right TMJ (purple arrow) explaining erosion or upper condylar head margin. Note no evidence of meningeal enhancement (orange) therefore no convincing intracranial extension. The diffuse nature of disease raises the possibility of an infective cause rather than neoplastic. (For interpretation of the references to colour in this Figure legend, the reader is referred to the web version of this article).

Initial tests showed WBC of 8.8 × 10^3^/L, C-reactive protein of 15.3 mg/L, HbA1C of 7.2 %, normal kidney function tests, and a negative HIV serology. Radiological assessment with CT head (Fig. 1) demonstrated thickening of the external auditory canal and middle ear with an ill-defined soft tissue mass pacifying the right mastoid air cells cavity. Subsequent MRI (Fig. 2 and Fig. 3) showed an extensive diffuse multi-compartmental enhancement with bone involvement suggestive of osteomyelitis.

A provisional diagnosis of MOE with SBO was made and the patient was commenced presumptively on antipseudomonal therapy in the form of parenteral then high dose oral ciprofloxacin therapy at 750 mg BID.

**Fig. 4:**
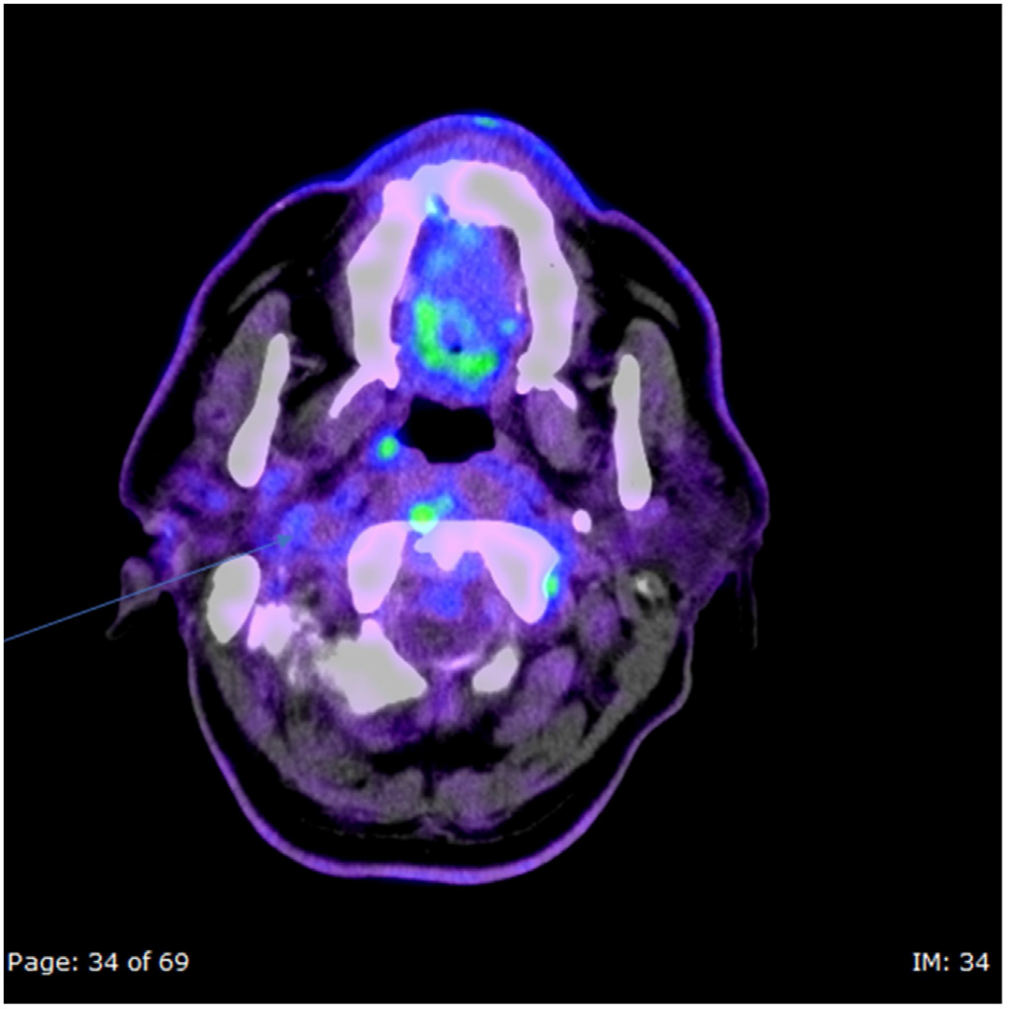
Axial FDG PET-CT demonstrates mild increased avidity in the right deep neck spaces including carotid and masticator spaces (arrow). The “low grade” nature of the tracer uptake suggests infective cause rather than malignant.

After 10 weeks of discharge, the patient represented with progression of symptoms complaining of worsening headache, dizziness, and double vision. Local examination showed swelling of the right ear canal and intact tympanic membrane with no accompanying discharge. In the view of disease progression while on treatment, PET -CT (Fig. 4) was arranged to rule out potential underlying neoplasms; but was furthermore supportive of an underlying infectious process. Subsequently, multiple microbiological and histological samples from the right ear canal, bone biopsies of the temporomandibular joint and soft tissues from the base of the skull demonstrated no evidence of malignancy or granulomas while extended culture yielded yeast eventually identified as *Candida orthopsilosis* matched from multiple sites. It was sensitive to amphotericin B (MIC 0.5 μg/mL) and fluconazole (MIC 0.5 μg/mL).

The patient was treated with two weeks induction course of liposomal amphotericin B (5 mg/Kg) followed by oral fluconazole (400 mg daily for a planned 6 months duration). Despite favorable initial response with the disappearance of the 6th nerve palsy, the patient had subsequent relapse with recurrent emergency visits and hospital admissions for worsening dizziness and vomiting. MRI was repeated and revealed persistent disease with increase in surrounding pachymeningeal enhancement. Accordingly, reinduction course of Amphotericin was considered followed by extended higher dose of fluconazole therapy of 800 mg daily scheduled for a prolonged duration of 12 months. During which, follow up MRI was repeated twice in 8 weeks interval, but showed no significant interval changes. One year following initial presentation, the patient is frail but stable.

## Discussion

Malignant Otitis Externa (MOE) is an aggressive and potentially fatal infection of the external ear canal, mastoid, and base of skull which was originally described by Chandler in 1968 [1]. The disease can progress into complicated skull base osteomyelitis where it can involve cranial nerves as well as extend to intracranial spaces [2]. The disease typically affects elderly patients with diabetes mellitus as well as immunocompromised such as patients with malignancies and HIV infection [3]. The reasons that patients with diabetes mellitus are susceptible to both bacterial and fungal infections at the ear canal is multifactorial; primarily due to poor tissues perfusion secondary to diabetic microangiopathy, higher cerumen pH as well as immune dysfunction predisposing to distinctive disease pathogens such as *Pseudomonas aeruginosa* [4]. Typical presenting symptoms are earache, ear discharge and unrelenting headache [5]. Anatomically, the spread of infection to temporal bone occur through the fissures of Santorini and tympanomastoid sutures, involving the stylomastoid and jugular foramina and eventually through venous channels and fascial planes to dural sinuses and petrous apexes towards brainstem stem with ominous consequences [6]

In 95 % of the cases of MOE, *Pseudomonas aeruginosa* is isolated as the main underlying pathogen but with better diagnostic techniques; other organisms have been identified such as *Staphylococcus aureus* particularly MRSA as well as other related gram negative bacteria such as *Klebsiella* and *Proteus species* [7]. Nevertheless, fungal infections are rare with infrequent case reports of *Aspergillus* and *Candida species.* Of note, it is difficult to establish the circumstances leading to acquisition of fungal MOE, but immune dysfunction and previous antibiotics use are probable contributing factors [6]. The most common etiological agents for fungal Skull Base Osteomyelitis (SBO) is *Aspergillus species* as highlighted in the previous reviews [8]. With better assessment and diagnostic techniques, *Candida species* is also becoming a recognized isolated pathogen(9)

Reviewing previously published cases, Hamzany et al., reviewed 60 patients with MOE diagnosed between 1990 and 2008 at a tertiary center, 9 of which (15 %) were caused by fungal etiology predominately *Candida species* mainly *Candida albicans* [9]. Interestingly, although the incidence of diabetes mellitus was comparable in patients with bacterial and fungal pathology, neurological complications were more frequent with the latter (14 % vs 55 %). Searching the literature for similar published case reports of fungal SBO in adults, yielded 9 cases, 6 of which were reported in English (Table1; (10–15)). Reported isolated pathogens are two of each; *Candida albicans*, *Candida parapsilosis* and *Candida glabrata*. Most of the patients were diabetic (5/6). Five out of six cases were treated successfully. Antifungal sensitivity tests were performed only in one patient (*Candida glabrata*: fluconazole susceptible, dose dependent) [10]. Duration of treatment varied between 3–6 months. Half of the patients received induction with amphotericin B or Echinocandins followed by azoles, and in two cases, oral fluconazole was given throughout the course. There was only one fatality; a renal transplant recipient succumbed to *Candida parapsilosis* MOE despite prolonged management with amphotericin B and flucytosine along with extensive surgery Table 1 [13].

**Table 1:** Previously reported cases of Candida SBO etiology, management, complications, and outcome.

Case no and reference	Patient details	Etiology	Complication	Procedure	Antifungal agent	Outcome
1 [11]	89, male	*Candida albicans*	Skull base extension leading to XII cranial nerve involvement	Biopsy of the ear canal	Anidulafungin 9 weeks, followed by oral Voriconazole for 6 months	Regression of osteomyelitis and resolution of XII cranial nerve at 6 weeks
2 [12]	60, male, Diabetes Mellitus	*Candida albicans*	Right sphenoid osteomyelitis	Biopsy of granulation tissue	Ora fluconazole. Duration of treatment not provided	Pain responded after 2 weeks of antifungal treatment
3 [15]	66, male, Diabetes Mellitus	*Candida glabrata*	sigmoid sinus thrombosis, left maxillary artery pseudoaneurysm, massive bleed	Endovascular embolization	Amphotericin followed by Caspofungin. Total duration 3 months	Good clinical improvement
4 [14]	87, male, Diabetes Mellitus	*Candida parapsilosis*	Extension to nasopharynx and paraesophageal space, facial palsy	Soft tissue biopsy only	Oral Fluconazole. Treatment duration not provided	Facial palsy resolved within 20 days of treatment
5 [10]	74, male, Diabetes Mellitus, End stage renal disease on Hemodialysis	*Candida glabrata*	Osteomyelitis of petrous bone	Right exploratory tympanotomy and mastoidotomy	IV Amphotericin for a total of 3 gm, followed by Fluconazole for 5 months	Improvement with Ear pain resolved within 20 days
6 [13]	58, male, Diabetes Mellitus, renal transplant recipient	*Candida parapsilosis*	Left XII cranial nerve and vocal cord palsy, Candidemia	Left subtotal petrosectomy	Amphotericin B plus Flucytosine. Patient expired while on treatment	Patient expired

From available limited evidence, following diagnosis of *Candida* MOE, prolonged antifungal therapy is warranted taking into consideration the associated SBO and potential central nervous system (CNS) involvement. Ideally, antifungal management should be guided by appropriate susceptibilities as well as bone and CNS penetration. While there are good supporting evidence for CNS penetration for amphotericin, flucytosine and azoles, there are limited data for echinocandins [16,17]. The IDSA recommendations for *candida* osteoarticular infection is surgical debridement, followed by lipid formulation amphotericin B or echinocandins for 2 weeks followed by a prolonged course of oral fluconazole for 6–12 months [18]. there is no specific mention of for fungal MOE or SBO in IDSA guidelines. Notably, surgery is no longer the mainstay of care for MOE except for bone biopsy and culture [8]. However, patients with more advanced disease may require debridement [19]. Our patient required prolonged treatment. He improved symptomatically and in terms of VIth nerve palsy, however radiologically remained stable disease.

## Conclusion

We present a rare case of fungal MOE leading to skull base osteomyelitis caused by *Candida orthopsilosis* which was diagnosed on aggressive investigation following failure to respond to initial antimicrobial therapy. We advocate that if there is disease progression in spite of antibiotic therapy, one should promptly evaluate for alternative pathology including fungal infections, verification of which necessitates microbiological and histopathological confirmation. Despite prolonged appropriate antifungal therapy, substantial morbidity and mortality has to be expected. To the best of our knowledge, this is the first case of *Candida orthopsilosis* MOE and skull base osteomyelitis case to be reported.

## Conflict of interest

No conflicts of interest.

## Sources of funding

Hamad medical corporation.

## Consent

Consent obtained from patient for case report publication

## Ethical approval

Obtained from institutional ethical board.

## Author contribution

Dr Junais Koleri – review of literature, collecting data, images, manuscript preparation

Dr Ahmed Bishawi – manuscript preparation

Dr Isra al shekh- review of literature

Dr Hamad abel hadi – review of literature, case preparation and analysis

Dr Salaman Qureshi – interpreting radiology, images

Dr Muna maslamani- manuscript preparation, guidance

## Compliance with ethical standards

Ethics approval and patients’ consent was obtained for the publication of this case reports and all accompanying images. Permission was obtained to publish the case reports from institutional review board which is in line with international standards.
